# Model checking: recent improvements and applications

**DOI:** 10.1007/s10009-018-0501-x

**Published:** 2018-07-24

**Authors:** Dragan Bošnački, Anton Wijs

**Affiliations:** Eindhoven University of Technology, Eindhoven, The Netherlands

**Keywords:** Model checking, Planning, Strategy synthesis, Probabilistic model checking, Partial-order reduction

## Abstract

Model checking (Baier and Katoen in Principles of model checking, MIT Press, Cambridge, 2008; Clarke et al. in Model checking, MIT Press, Cambridge, 2001) is an automatic technique to formally verify that a given specification of a concurrent system meets given functional properties. Its use has been demonstrated many times over the years. Key characteristics that make the method so appealing are its level of automaticity, its ability to determine the absence of errors in the system (contrary to testing techniques) and the fact that it produces counter-examples when errors are detected, that clearly demonstrate not only that an error is present, but also how the error can be produced. The main drawback of model checking is its limited scalability, and for this reason, research on reducing the computational effort has received much attention over the last decades. Besides the verification of qualitative functional properties, the model checking technique can also be applied for other types of analyses, such as planning and the verification of quantitative properties. We briefly discuss several contributions in the model checking field that address both its scalability and its applicability to perform planning and quantitative analysis. In particular, we introduce six papers selected from the 23rd International SPIN Symposium on Model Checking Software (SPIN 2016).

## Introduction

The current issue of the journal Software Tools for Technology Transfer (STTT) contains six revised and extended versions of papers presented at the 23rd International SPIN Symposium on Model Checking Software (SPIN 2016) [8]. SPIN 2016 was held in Eindhoven, The Netherlands, on 7–8 April 2016 collocated with the Joint European Conferences on Theory and Practice of Software (ETAPS). These six papers were selected by the guest editors out of the sixteen papers presented at the event, based on their ranking given by the peer reviewers.

During the last two decades the SPIN symposiums have established themselves as traditional annual forums for researchers and practitioners for the verification of software systems. The evolution of the SPIN events has to a great extent mirrored the maturing of model checking into a prevailing technology for the formal verification of software systems. The first SPIN workshop was held in Montreal in 1995. The next couple of subsequent editions of SPIN were intended as gatherings for presenting extensions and applications of the model checker Spin  [24], to which the series owes its name. Starting with the 2000 edition, the scope of the event clearly broadened to include techniques for formal verification and testing in general. In addition, the SPIN events aim to promote interaction and exchange of ideas across related software engineering areas, like static and dynamic analysis.

This special issue nicely demonstrates the current scope of the SPIN events. First of all, in addition to the Spin model checker, contributions in this issue use the tool TAPAAL [13], the Afra model checking tool [29], the ASSET tool [40], and the Cadp toolbox [19].

Second of all, the majority of the papers in this issue are on extending and applying model checking beyond its traditional set-up, i.e. the formal verification of concurrent systems w.r.t. qualitative behavioural properties. Four of the six papers are on the application of model checking to construct a strategy or plan to solve a particular scheduling or control problem constrained by time and/or resource requirements. Another paper is on on-the-fly verification of *quantitative* properties via probabilistic model checking [3]. In that sense, one of the papers is more traditional in its scope, but it addresses the main drawback of model checking, i.e. its limited scalability, by contributing to the topic of partial-order reduction [22, 35, 39], a very effective technique to mitigate state space explosion.

The remainder of this preface is organised as follows: Section 2 discusses the use of model checking for the synthesis of strategies and plans. In Sect. 3, the verification of quantitative properties by means of probabilistic model checking is considered. Partial-order reduction to on-the-fly reduce state spaces explored by model checkers is discussed in Sect. 4. Finally, in Sect. 5, some concluding remarks are given.

## Planning and strategy synthesis

The application of model checking to construct a plan or synthesise a strategy is not far-fetched, as model checking and planning have much in common [1, 11, 37, 43, 44]: in both cases, a (large) state space has to be explored, looking for interesting behaviour. While in traditional model checking, this behaviour is essentially undesirable, violating some functional properties, in planning the interesting behaviour is desirable and constitutes a successful plan to optimise a system while fulfilling given constraints. When synthesising a strategy, typically the notion of a controller is added to the model, and the question is whether there exists a strategy for that controller such that any possible behaviour under that strategy satisfies the specification.

In the paper *Integrating river basin DSSs with model checking* by del Mar Gallardo et al. [18], which extends their SPIN 2016 paper [17], it is demonstrated how the Spin model checker can be applied in a decision support system (DSS) that mitigates the effects of floods in river basins. Model checking is used to synthesise management recommendations that meet the constraints given by the dam manager. A set of constraints is added to a Promela model that interacts with an external model for the river basin. Spin exhaustively explores all possible manoeuvres and produces a trace, i.e. a sequence of manoeuvres, that fulfils the given constraints.

The paper *A Case Study of Planning for Smart Factories – Model Checking and Monte-Carlo Search for the Rescue* by Edelkamp and Greulich [15], which extends their SPIN 2016 paper [16], proposes to use the Spin model checker to construct plans for multi-agent systems that control the industrial production of goods. Assembling stations use queues to buffer materials, and the core objective is to optimise the throughput of the system. The authors demonstrate that by using branch-and-bound searching, optimised plans consisting of thousands of steps can be produced in reasonable time. For comparison, they also consider using a Monte Carlo search framework and conclude that such an approach is even better in constructing plans. They conjecture that building a model checker that uses Monte Carlo search is an interesting topic to investigate in future work.

Of course, timing is crucial when synthesising strategies to control real-time systems, but its introduction makes the use of model checking more challenging. The previous contribution handles timing by carefully modelling it explicitly such that a model checker unaware of timing could still be used. An alternative is to use model checking techniques that natively support timing. Symbolic continuous-time on-the-fly methods, such as those employed in the tools Kronos [9], UPPAAL [5], Tina [6] and Romeo [20], have been employed in on-the-fly algorithms for controller synthesis [4, 36]. However, for such a task, discrete-time methods turn out to be very competitive [2].

The paper *Discrete and Continuous Strategies for Timed-Arc Petri Net Games* by Jensen et al. [25], which extends their SPIN 2016 paper [26], addresses this topic and proposes an on-the-fly algorithm for the synthesis of timed controllers relative to safety objectives. It turns out that when restricting the context to the use of urgent controllers that act immediately or wait for another occurrence of the same event, then discrete-time methods can be used to determine the existence of a continuous-time safety controller.

Schedulability and resource utilisation of wireless sensor and actuator network (WSAN) applications are addressed in the paper *Modeling and Analyzing Real-Time Wireless Sensor and Actuator Networks Using Actors and Model Checking* by Khamespanah et al. [27]. This paper extends their SPIN 2016 paper [28]. Such applications can be modelled by defining a number of concurrent actors, each providing services that can be requested by other actors by sending messages. Schedulability of the operations can be checked using Timed Rebeca, and Timed Computation Tree Logic (TCTL) model checking can be performed to check more complicated properties, such as minimal resource utilisation.

## Probabilistic model checking

To check quantitative properties of systems, for example referring to time constraints or energy consumption, models can be extended with probabilities associated with behavioural events. The potential behaviour of such systems can then be captured in Markov Chains or probabilistic transition systems (PTSs) [21], which essentially are discrete-time Markov Chains in which transitions are labelled with actions and probabilities, and communication between concurrent processes is modelled. Probabilistic model checkers, such as Prism  [30] and Storm  [14], can be used to analyse these Markov Chains and determine whether they satisfy given probabilistic properties. To express these properties, suitable temporal logics need to be defined, such as probabilistic computation tree logic (PCTL) [23].

In the paper *On-the-Fly Model Checking for Extended Action-Based Probabilistic Operators* by Mateescu and Requeno [32], which extends their SPIN 2016 paper [33], a new regular probabilistic operator is proposed to specify the probability measure of a path described by a generalised regular formula involving computations on data values. This operator subsumes the until operators of PCTL and their action-based counterparts. The authors integrate this operator into MCL (Model Checking Language) and implement an on-the-fly model checking method in the CADP toolbox.

## Partial-order reduction

The partial-order reduction (POR) technique [22, 35, 39] is perhaps the most efficient technique to mitigate the state space explosion problem in model checking. In recognition of this fact the founding fathers of POR, Godefroid, Peled, Valmari, and Wolper, received the 2014 CAV award. POR exploits the observation that the state space may contain several paths that are similar, in the sense that their differences are not relevant to the property under consideration. By pruning certain transitions, the size of the state space can be reduced.

The current issue features the paper *Fair Testing and Stubborn Sets* by Valmari and Vogler [41], which extends their SPIN 2016 paper [42]. Valmari was the first to notice the necessity for the so-called *cycle proviso* to ensure the correctness of POR when cycles are present in the state space. In the presence of cycles, POR without such a proviso may incorrectly terminate after having investigated a cycle, consistently ignoring behaviour that leaves the cycle. Hence, this problem is known as the *ignoring problem*. The cycle proviso turned out to be crucial for various adaptations of POR to different search orders of the state space (such as breadth-first search [7]), as well as parallel searches, both for shared memory (in settings using multiple cores [31] and graphics processing units [34]) and distributed architectures [10, 38].

In the paper by Valmari and Vogler, it is proven that a partial-order method originally proposed for trace equivalence also preserves fair testing equivalence, in which deadlocks are unified with livelocks that cannot be exited. Thus, it supports a practical fairness assumption. Compared to the original SPIN 2016 paper, the extended version presents new observations regarding the ignoring problem in this context, remarking that the preservation of trace and fair testing equivalence does not imply that the ignoring problem is addressed.

## Conclusions

Recent improvements and applications in the field of model checking have been discussed and associated with six papers selected from SPIN 2016, that have been included in this special issue. Four of the six papers contribute work on the application of model checking techniques to construct schedules and plans for planning problems, and synthesise strategies for control problems. In addition, one paper contributes to the verification of quantitative properties, and one contributes to the topic of partial-order reduction. Together, these papers address both the strengthening of the model checking method itself and its applicability to efficiently solve problems outside its traditional scope.
